# mRNA in exosomas as a liquid biopsy in non-Hodgkin Lymphoma: a multicentric study by the Spanish Lymphoma Oncology Group

**DOI:** 10.18632/oncotarget.16435

**Published:** 2017-03-22

**Authors:** Mariano Provencio, Marta Rodríguez, Blanca Cantos, Pilar Sabín, Cristina Quero, Francisco R. García-Arroyo, Antonio Rueda, Constanza Maximiano, Delvys Rodríguez-Abreu, Antonio Sánchez, Javier Silva, Vanesa García

**Affiliations:** ^1^ Department of Medical Oncology, IDIPHIM, University Hospital Puerta de Hierro Research Institute, Madrid, Spain; ^2^ Department of Medical Oncology, Gregorio Marañon Hospital, Madrid, Spain; ^3^ Department of Radiotherapy, Virgen de la Victoria Clinic University Hospital, Málaga, Spain; ^4^ Department of Medical Oncology, Pontevedra Hospital, Pontevedra, Spain; ^5^ Department of Medical Oncology, Costa del Sol Hospital, Marbella, Spain; ^6^ Department of Medical Oncology, Gran Canaria University Hospital, Las Palmas de Gran Canaria, Spain; ^7^ Molecular Oncology Laboratory, IdISSC, Clinico San Carlos University Hospital, Madrid, Spain

**Keywords:** exosomes, mRNA, liquid biopsy, B-cell lymphomas, BCL-6

## Abstract

**Purpose:**

To determine the feasibility of mRNAs (*C-MYC, BCL-XL, BCL-6, NF-κβ, PTEN* and *AKT*) in exosomes of plasma as a liquid biopsy method for monitoring and prognostic evolution in B-cell lymphomas.

**Patients and Methods:**

Exosomes were isolated from 98 patients with B-cell Lymphoma and 68 healthy controls. mRNAs were analyzed by quantitative PCR. An additional 31 post-treatment samples were also studied.

**Results:**

In the general and follicular lymphoma series, the presence of *AKT* mRNA was associated with poor response to rituximab-based treatment. Patients with first relapse or disease progression showed a lower percentage of *PTEN* and *BCL-XL* mRNA. The presence of *BCL-6* mRNA was associated with a high death rate. The absence of *PTEN* mRNA in the general series, and presence of *C-MYC* mRNA in follicular lymphomas, were associated with short progression-free survival. *BCL-6* and *C-MYC* mRNA were independent prognostic variables of overall survival. *C-MYC* mRNA may provide prognostic information with respect to overall survival. *BCL-XL* mRNA and increase of *BCL-6* mRNA in post-treatment samples could serve as molecular monitoring markers.

**Conclusions:**

This is the first large study to evaluate the prognostic and predictive values of pretreatment tumor-associated mRNA in exosomes. BCL-6 and C-MYC mRNA positivity in pretreatment samples were predictors of worse PFS compared to patients with mRNA negativity. C-MYC mRNA positivity was also a statistically significant predictor of inability to obtain complete response with first-line therapy.

## INTRODUCTION

Follicular lymphoma (FL) and Diffuse Large B-Cell Lymphoma (DLBCL) account for over 70% of all non-Hodgkin Lymphomas (NHL) and rituximab is approved for the treatment of these common subtypes [1]. The identification of several lymphocyte-specific surface antigens has improved the possibility of developing effective and relatively specific targeted therapy for NHL.

The prognosis for patients with primary resistant or relapsed aggressive lymphomas is still poor as over 20% of patients with DLBCL die and 50% relapse within two years of diagnosis. In the case of FL, around 50% will have relapsed at 5 years even when treated with chemotherapy *plus* rituximab. Currently, we have no molecular prognostic indicators and only known clinical factors are used.

In agreement with Roschewski et al., we believe that assays of circulating tumor RNA/DNA could serve as “liquid biopsies” thereby providing information regarding response or resistance to treatment, in addition, this technique could be of use in both follicular and aggressive lymphomas [2].

Exosomes are nanovesicles not exceeding 100 nanometers protected by a lipid-rich bound membrane. They develop in the cytoplasm, specifically the endoplasmic reticulum, in a variety of normal and pathological cells, including tumor cells. Their differing cells of origin influence their protein content, cytoplasmic or membrane proteins and genetic material. This genetic material can be used to perform liquid biopsies to analyze prognostic and predictive biomarkers. From a functional standpoint, this genetic material, mRNA and miRNA, can epigenetically reprogram recipient cells [3], in addition to having other functions related to evasion of host immune response against the tumor [4]. One challenge is to determine how exosomes work *in vivo*, and ascertain whether current information obtained from *in vitro* studies can be used to understand the mechanisms developed by exosomes within tumors.

The potential use in clinical practice of mRNA presence in exosomes of plasma from patients with B-Cell Lymphoma, which could be taken as liquid biopsies, and their applicability to all NHL subtypes remains to be defined, and especially with respect to predicting prognoses, response to treatment and monitoring during follow-up. In this study, mRNA presence of *C-MYC, BCL-XL, BCL-6, NF-κβ, PTEN* and *AKT* in exosomes is analyzed.

The six genes evaluated in this study have important roles in the deregulated pathways of many cancers, including lymphoid neoplasms. The PI3K/AKT/mTOR and NF-κβ pathways are involved in several steps of tumorogenesis, such as cell proliferation, survival, angiogenesis and tumor cell drug/radiation resistance [5–7]. AKT regulates effectors with roles in cell survival, for example BCL-2 and NF-κβ, which are also dysregulated in B-Cell lymphomas [5]. Unfavorable clinical characteristics in DLBCL have been strongly associated with NF-κB pathway [8, 9]. In addition, PTEN is a negative regulator of the PI3K/AKT pathway [7]. BCL-xl expression is regulated by the NF-kB and AKT pathways, and rituximab treatment led to downregulation of BCL-XL expression by inhibiting NF-kB DNA-binding activity [10]. With regard to BCL6, this prevents apoptosis and has been reported to predict survival in patients with diffuse large-B-cell lymphoma [11]. However, in the post-rituximab era, there is controversy about the prognostic value of BCL6 expression in B-cell lymphomas. Finally, C-MYC is a potent oncogene which is also involved in cycle cell activation and, in lymphoma patients, is a predictor of more aggressive clinical behavior and poor response to therapy [12, 13]. On basis of the above, the analysis of these mRNAs in liquid biopsies could have both prognostic and predictive values.

## RESULTS

### General characteristics of patients

A total of 98 patients with follicular or diffuse large B-cell lymphoma were recruited for this study. Patient characteristics are shown in Supplementary Table 1. In the series studied, 53% were male, median age 60 years (range 26-87), 31% had B symptoms and 45% had extra nodal involvement. When the population was classified on the basis of risk factors, 26% and 27% were grouped into high risk (International Prognostic Index (IPI) and Follicular Lymphoma International Prognostic Index (FLIPI), respectively).

### Efficacy and survival results

The overall response rate (ORR) was 93%, complete response (CR) was obtained in 67% patients, and 26% achieved partial remission (PR). After a median follow up of 28 months (range: 19-103 months), 18% of patients relapsed and 15% died.

When comparing progression-free survival (PFS) and overall survival (OS) between FL and DLBCL patients, no significant differences were found between these groups (Supplementary Table 2). As a result, survival analysis was also performed in the general series which, at 36 month follow-up, demonstrated a PFS of 80.2% (95% CI, 71.4-89%) and OS of 85.2% (95% CI, 77.4-93%).

### Presence of mRNA in exosomes

Exosomes were quantified by acetylcholinesterase activity, and were detected in plasma from DLBCL and FL patients. No differences in exosome levels were found between the lymphoma groups studied and healthy controls. Levels of acetylcholinesterase activity are show in Supplementary Figure 1. Exosome levels were not associated with response to treatment or patient outcomes.

The studied mRNAs were detected in exosomes in the following proportions: 9% for *C-MYC*, 16% for *BCL-XL*, 34% for *BCL-6*, 8% for *NKkB*, 25% for *PTEN* and 3% for *AKT*. *BCL-6* mRNA was detected in more patient plasma samples than in plasma from healthy controls (*p*<0.001, χ^2^ test), while presence of *PTEN* mRNA was detected in more control cases (*p*<0.001, χ^2^ test). In addition, the frequency of positivity for *BCL-6* mRNA in plasma was higher in DLBCL than in FL patients (*p*=0.005, χ^2^ test), and presence of *BCL-XL* was lower in DLBCL (*p*=0.024, χ^2^ test). The frequencies of each mRNA in DLBCL, FL and controls are shown in Supplementary Table 3. In addition, a higher number of mRNAs detected in the same patient was associated with a lymphoma subpopulation and mainly DLBCL (Supplementary Table 4).

### mRNA in exosomes from plasma and patient survival

Patients with first relapse or disease progression during follow-up had a lower percentage of *PTEN* and *BCL-XL* mRNA presence in exosomes at the time of diagnosis (*p*=0.042 and *p*=0.049, respectively, χ^2^ test). This association with *PTEN* mRNA was also found in the DLBCL subpopulation (*p*=0.038, χ^2^ test). Data of these associations are showed in Table 1A. Furthermore, in the general series, a trend toward significant association was observed between the presence of *BCL-6* mRNA at the time of diagnosis and a high patient death rate (*p*=0.052, χ^2^ test, Table 1B).

Table 1Associations found between outcome of disease and evaluated mRNA in exosomes, either in total series (B-cells) and subpopulations (DLBCL and FL)

**Table:** 

A	PTEN (B-cells)			PTEN (DLBCL)			BCL-XL (B-cells)		
	positive	negative	*p*	positive	negative	*p*	positive	negative	*p*
**Relapse/Progression**									
No	24.2	57.9	0.042	24.1	56.9	0.038	15.8	66.3	0.049
Yes	1.1	16.8		0.0	19.0		0.0	17.9

**Table:** 

B	BCL-6 (B-cells)		
	positive	negative	*p*
**Exitus**			
No	26.0	59.4	0.052
Yes	8.3	6.3

**Table:** 

C	AKT (B-cells)			AKT (FL)		
	positive	negative	*p*	positive	negative	*p*
**Response to treatment**						
Response	2.1	91.5	0.052	2.8	88.9	0.028
Non-response	1.1	5.3		2.8	5.6	

Data are shown in percentages.

#### Progression-free survival

**Figure 1:**
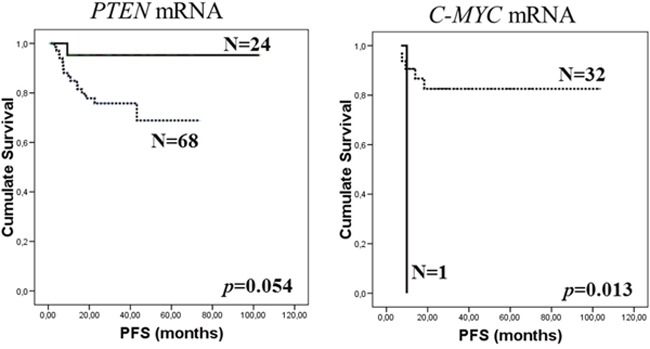
Kaplan-Meier PFS curves in relation to presence of *PTEN* mRNA in total series **(A)** and *C-MYC* mRNA in FL series **(B)** in exosomes of plasma from pretreatment samples. Continuous curves show cases with mRNA absence and discontinuous curves show cases with mRNA presence.

In the general series, the Kaplan-Meier test showed a trend to significant association between the absence of *PTEN* mRNA in exosomes and patients with a short PFS (*p*=0.054; Figure 1A). When the follicular series were analyzed alone, there was a significant association between the presence of *C-MYC* mRNA in exosomes and PFS (*p*=0.013, Kaplan-Meier test; Figure 1B). At 36-month follow-up, PFS was 95.2% and 0% (95% CI, 86.1-100% and 0-0%) in patients with presence of *PTEN* and *C-MYC* mRNA, respectively, while PFS was 74.9% and 82.6% (95% CI, 63.8-86.1% and 68.5-96.7%) in patients without. Adjusted analysis showed no associations between the mRNAs analyzed and PFS.

#### Overall survival

**Figure 2:**
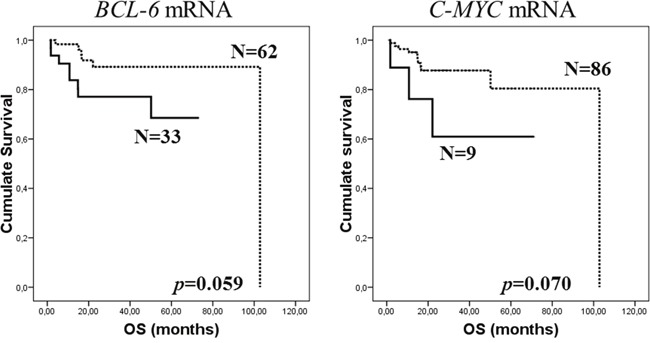
Kaplan-Meier OS curves in relation to presence of *BCL-6* **(A)** and *C-MYC* **(B)** mRNA in exosomes of pretreatment plasma samples of total series. Continuous curves show cases with mRNA absence and discontinuous curves show cases with mRNA presence.

When the general series were analyzed, a trend toward significant difference was observed in OS for the presence of *BCL-6* and *C-MYC* mRNA in exosomes: patients with these mRNAs present in plasma at diagnosis had shorter survival than patients without (*p*=0.059 and *p*=0.070, respectively, Kaplan-Meier test; Figure 2). At 36-month follow-up, patients with *BCL6* and *C-MYC* mRNA had OS of 77.1% and 60.9% (95% CI, 62.2-92% and 25.5-96.3%, respectively), while patients without had OS of 89.2% and 87.7% (95% CI, 80.2-98.2% and 80.1-95.3%, respectively). No significant differences in OS were found, including in the two subpopulations, with the other mRNAs analyzed.

Unadjusted analysis was performed to determine the influence of mRNA in plasma and the clinicopathological parameters in OS. Clinicopathological variables that could be regarded as statistically supported factors in OS prediction were: stage, extranodal involvement, IPI, response to rituximab-based treatment and first relapse. The multivariate Cox's regression model determined, as independent prognostic variables for OS, the presence or absence of *BCL-6* or *C-MYC* mRNA in exosomes, together with occurrence of the first relapse/progression (Table 2A).

Table 2Multivariate Cox analysis of the association between presence of MYC and BCL-6 mRNA in exosomes and OS of lymphoma cancer patients (A). Statistical interaction between MYC mRNA variable and tumor pathological stage for predicting OS outcome value in lymphoma patients (B)

**Table:** A

Variable	Category	HR	95% CI	*p* value
First relapse/Progression	Yes vs No	10.862	2.672-44.159	0.001
MYC mRNA	Presence vs absence	8.519	1.819-39.887	0.007
BCL-6 mRNA	Presence vs absence	9.095	1.706-48.476	0.010

**Table:** B

Variable	Category	HR	95% CI	*p* value
RT	Complete vs non-complete	7.706	2.251-26.377	0.001
RT*C-MYC mRNA	RT*Presence *C-MYC*	9.980	1.707-58.334	0.011

RT: response to treatment

#### Exosome-related mRNA in plasma and survival in the series stratified according to response to treatment

As stated above, in our general series recruited before receiving rituximab-based treatment, 93% of the lymphoma patients showed complete or partial response to rituximab-based chemotherapy. In this series, the presence of *AKT* mRNA in exosomes was significantly associated with non-response to rituximab-based treatment (*p*=0.052, χ^2^ test). This association was also observed in the follicular lymphoma subpopulation (*p*=0.028, χ^2^ test), but not in DLBCL. Additional details are included in Table 1C.

**Figure 3:**
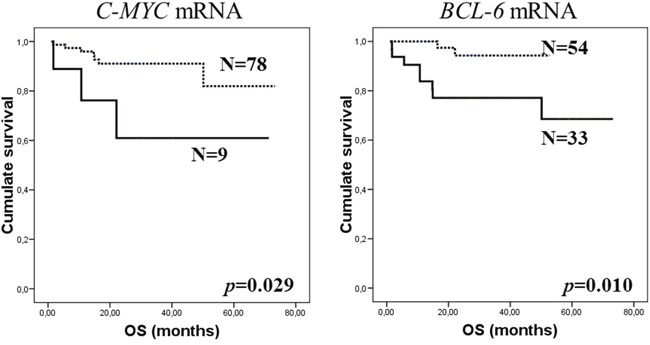
Kaplan-Meier OS curves in relation to presence of *C-MYC* **(A)** and *BCL-6* **(B)** mRNA in exosomes of pretreatment plasma samples from responsive patients group to rituximab-based chemotherapy. Continuous curves show cases with mRNA absence and discontinuous curves show cases with mRNA presence.

In addition, we evaluated the prognostic value of mRNA in the series stratified according to response to treatment. In patients who responded to rituximab-base treatment, the presence of *AKT* mRNA in exosomes was associated with worse PFS (*p*=0.038). The Kaplan-Meier analysis also showed that presence of *C-MYC* and *BCL-6* mRNA correlated with worse OS in the responsive patient group (*p*=0.029 and *p*=0.010, respectively, Figure 3). When the Cox's regression models were assessed in stratified series, *C-MYC* mRNA interacted with response to treatment, giving a prognostic value for OS in non-responsive patients (Table 2B).

### Presence of exosome-related mRNA in plasma from patients after treatment with rituximab-based chemotherapy

Our molecular variables were analyzed in exosomes obtained from plasma of 31 patients in our series after rituximab-based treatment. *C-MYC* mRNA was detected in 6 post-treatment samples, *BCL-XL* in 3 cases, *BCL-6* in 10 and *PTEN* in 6; and no samples were positive for *NFkB* or *AKT*. Differences in the mRNA detection between pre- and post-treatment samples from the same patient are shown in Supplementary Table 5.

In this subgroup of post-treatment samples, the presence of *BCL-XL* mRNA was associated with a high death rate (*p*=0.046, χ^2^ test) and, when only the FL subpopulation was analyzed, a significant association was observed between the presence of *BCL-6* mRNA and progression (*p*=0.047, χ^2^ test). More details are shown in Table 3A. When changes were observed between pre-treatment and post-treatment samples, an association was detected: new detection or increase of *BCL-6* mRNA in post-treatment samples compared to pre-treatment samples was associated with non-response to treatment (*p*=0.038, χ^2^ test, Table 3B).

Table 3Associations found between outcome of disease and evaluated mRNA in exosomes from post-treatment samples

**Table:** 

A	BCL-XL (B-cells)				BCL-6 (FL)		
	positive	negative	*p*		positive	negative	*p*
**Exitus**				**Response to treatment**			
No	6,5	87,1	0.046	Response	14,3	78,6	0.047
Yes	3,2	3,2		Non-response	7,1	0

**Table:** 

B	Response to treatment		
	Response	Non-response	*p*
**BCL-6 (B-cells)**			
Absence in both samples	48.4	3.2	0.038
Decreased in post-treatment samples	32.3	0.0	
Increased in post-treatment samples	9.7	6.5	

Data are shown in percentages

## DISCUSSION

This is the first large *in-vivo* study to evaluate the prognostic implications of pretreatment tumor-associated exosomal mRNA. BCL-6 and C-MYC mRNA positivity in pretreatment samples was seen to be a statistically significant predictor of worsened PFS compared to patients with mRNA negativity. C-MYC mRNA positivity was also a statistically significant predictor of inability to obtain CR with first-line therapy.

Our work was based on a previous study [14] involving a different group of patients in whom we observed that, in lymphomas, it was feasible to identify the presence of exosomes in blood, and that unfavorable markers in plasma, mainly *C-MYC* mRNA, were associated with poor prognosis in B-cell lymphomas,. The latter was subsequently ratified in tissue series [15, 16].

We attempted to identify risk populations through an easily accessible source that could also be evaluated in evolutional terms.

To date, one publication has analyzed exosome production and release from aggressive B-cell lymphoma cells *in vitro* and *in vivo* [17]. Our clinical study analyzed two large populations, namely follicular and diffuse lymphoma patients. Given that, at present, the two groups share overlapping treatments, at least in first-line treatment, we analyzed the results and found that overall and disease-free survival during the observation period were similar in both groups. This led us to analyze all the liquid biopsies to determine whether there were any common factors that linked the two groups. We believe that ours is the first study to analyze this question in patients. The results show that the presence of *BCL-6* mRNA is significantly associated with a high risk of death in B-cell lymphomas.

In our series, the data on the exosomes obtained from the plasma of 31 patients after undergoing treatment are of particular interest. In this sub-group of post-treatment samples, the presence of *BCL-XL* mRNA was associated with a high death rate. Moreover, the presence of *BCL-6* mRNA in post-treatment samples from patients who did not exhibit this mRNA in pre-treatment samples was associated with non-response to treatment. These findings stimulate thought about the value of monitoring molecular response and the future development of intensification protocols or drugs with no crossed resistance *versus* conventional treatments in patients who present resistance. The simple and accessible identification of patients with initial resistance to treatment, without the need for aggressive maneuvers, would provide a magnificent tool for future studies.

We also found a significant association between presence of *C-MYC* mRNA in exosomes and short PFS only in FL. A significant association was observed between the presence of *BCL-6* mRNA in post-treatment samples and progression as response to treatment when only the FL subpopulation was analyzed. Larger series will be required to confirm these findings, however they open the way to new perspectives since they would allow better selection of patients who are suitable for consolidation therapies with radioimmunoconjugates, maintenance therapy or new drugs.

When we analyzed the complete series and studied overall survival, the presence of *BCL-6* and *C-MYC* mRNA in exosomes was associated to shorter survival and there were no differences in the other mRNAs analyzed. *C-MYC* remained as an independent prognostic variable for overall survival when stratified by response.

Different immunohistochemical algorithms are currently being tested with varying results among patient series [18, 19] but, to our knowledge, there are no serum or exosome studies which contribute to establishing prognoses or monitoring response in lymphomas. Our study could well be even more relevant in relation to the determination of BCL-6 through immunohistochemistry despite the fact that staining results varied dramatically [17].

In general series, the presence of *AKT* mRNA in exosomes was associated with non-response to rituximab-based treatment. In addition, we evaluated the prognostic value of mRNA in the series stratified according to response to treatment. In patients who responded to treatment, the presence of *AKT* mRNA in exosomes was associated with worse PFS. Preclinical data have been published that support our results, in that the inhibition of the AKT pathway by rituximab inhibits NFkB activity and the function and expression of BCL-XL. The role of the AKT pathway in the regulation of resistance to rituximab was corroborated by the use of an AKT inhibitor that succeeded in reversing resistance to rituximab in cell lines [20]. Our research also adds possible therapeutic relevance and we might be able to design protocols that incorporate AKT inhibitors that would revert any theoretical resistance to rituximab. Development of targeted agents for the treatment of DLBCL includes clinical evaluation of enzastaurin, an agent that suppresses signaling through protein kinase C and AKT pathways. NFkB can be inhibited indirectly with bortezomib. Some encouraging results were obtained in a phase II trial of bortezomib plus chemotherapy in patients with relapsed or refractory DLBCL [21]. All the above imply that there is an interconnection of the AKT pathways, NFkB, BCL-XL and PTEN in the mechanisms of resistance to rituximab.

Our research provides exosomal tumor markers which identify a clear population for further studies. One clinical application could be the design of a dose-intensified chemotherapy clinical trial in high risk patients, in the middle or after standard chemotherapy. Another would be the assessment of response when it is not clearly defined in imaging tests, to select high risk patients to perform a biopsy. In addition, the ease and reproducibility of the determination means that large amounts of biopsy material are not required and liquid biopsies can easily be combined with PET-TAC to evaluate the correlation between this imaging technique and serum data.

## MATERIALS AND METHODS

### Patients and clinical characteristics

A total of 98 patients (60 DLBCL, 38 FL) were recruited between January 2005 and January 2011 and followed-up clinically until November 2014. The study was conducted within the GOTEL Group (details can be seen in the Supplementary Methods section). Samples were provided by the 7 participating Spanish hospitals, and molecular analyses were centralized in Puerta de Hierro University Hospital. Written informed consent was obtained from all participants after an explanation of the nature of the study as approved by the research ethics board of the seven hospitals. Histological diagnoses were ascertained by lymph node biopsy. The WHO Classification histological criteria were used for sample diagnosis and classification [22]. Clinical and laboratory data (supplementary methods) were available at the time of diagnosis.

Blood samples were taken before beginning treatment and, between 4-6 weeks after treatment, blood samples were taken from 16 DLBCL patients and 15 FL. Blood samples were also obtained from 68 healthy blood donors.

### Clinical follow-up and treatment

Prospective follow-up was based on regular clinical, biochemical and radiological examinations, gallium scan and nuclear magnetic resonance or positron emission tomography if recommended by radiologists. In total, 86% of patients received 6 cycles of R-CHOP every 21 days. The remaining patients received rituximab-based treatment with variations of this combination. Response to treatment was assessed on the basis of clinical, radiological and pathologic criteria, according to modified International Workshop Response Criteria Guidelines [23]. Details are shown in supplementary data.

### Isolation and quantification of exosomes

Plasma was prepared by centrifugation of peripheral blood at 2,500 rpm for 25 minutes. Exosomes were isolated from 3 ml of plasma by differential centrifugations. To quantify the amount of exosomes we measured the activity of acetylcholinesterase. Methodological details have been described previously [24].

### RNA extraction and real-time PCR

RNA was extracted using the mirVana™miRNA Isolation Kit (Ambion Inc., TX) and quantified by NanoDrop ND-1000 (Thermo Scientific, DE). RNA was retro-transcribed with the Gold RNA PCR Core Kit (PE Applied Biosystems, CA) using random hexamers, in line with the manufacturer's instructions. Real-time PCR was performed in duplicate in a Light-Cycler apparatus using the LightCycler 480 SYBR Green I Master Kit (Roche Diagnostics, Germany), in line with the manufacturer's instructions. Additional details are shown in Supplementary methods and Supplementary Table 6. In a previous study [16], 3 common housekeeping genes in plasma led to erroneous normalization, which could have been explained by the active release mechanism of exosomes. For this reason, we analyzed the target mRNAs as presence or absence of mRNA in exosomes.

### Data analysis

The clinicopathological parameters were contrasted with the presence of target mRNAs by the χ^2^ test. The relationship between the cumulative probability of survival, as well as analyzed predictors, was calculated by the Kaplan-Meier method. Significant differences between curves were evaluated with Mantel's log-rank test. To identify factors that might be of independent significance in influencing survival, the Cox proportional risk regression model was applied. Box plot was performed applying logarithm of data. *p* values ≤0.05 were considered statistically significant. Statistical analysis was performed using the SPSS software, version 14.0 (SPSS Inc. IL).

## SUPPLEMENTARY MATERIALS FIGURES AND TABLES


