# Supplementary material for: mRNA in exosomas as a liquid biopsy in non-Hodgkin Lymphoma: a multicentric study by the Spanish Lymphoma Oncology Group
Source: Oncotarget. 2017 Mar 22;8(31):50949–57. doi: 10.18632/oncotarget.16435 (PMC5584220; doi:10.18632/oncotarget.16435)
Supplement: Supplementary file 1 [file oncotarget-08-50949-s001.pdf]

mRNA in exosomas as a liquid biopsy in non-Hodgkin Lymphoma: a multicentric study by the Spanish lymphoma oncology group

SUPPLEMENTARY DATA

Patients and clinical characteristics

The study was conducted within the GOTEL Group, the Oncology Group for the Treatment and Study of Lymphomas, a cooperative group set up within the Spanish Society of Medical Oncology by a network of Medical Oncology Services in Spain. Information regarding the objectives, structure, statutes and participating centers can be found on GOTEL's web site, www.grupolinfomas.es

Cases associated with HIV or HVC infections, or previous immunosuppressive treatments, were excluded. All histological samples corresponded to initial diagnostic biopsies prior to treatment. All cases stained positively for CD20. Cases diagnosed as T-cell histiocyte-rich B-cell lymphoma, primary mediastinal B-cell lymphoma, cutaneous LBCL and intravascular LBCL were excluded.

The following clinical and laboratory data were available: age, gender, stage (Ann Arbor system), histological grade, "B" symptoms, number of affected lymph node regions, number of extranodal sites, bone marrow infiltration, performance status, bulky disease, detection of Hepatitis C Virus, levels of lactate dehydrogenase, β_2-microglobulin and albumin and standard hematological tests. International Prognostic Index (IPI) and FLIPI (for FL) scores were determined.

Clinical follow-up and treatment

Prospective follow-up, starting after diagnosis and treatment, was based on a regular (every 3 months during the first and second year, every 6 months during the third year and then yearly until relapse) clinical, biochemical and radiological examination (chest and abdominal CT), gallium scan and MNR or PET if recommended by radiologists. R-CHOP consisted of cyclophosphamide, doxorubicin, vincristine, prednisone and rituximab, administered according to the standard doses for this regimen. Complete response (CR) was defined as the complete disappearance of any radiological or biologic lesion present at diagnosis and absence of new lesions. Partial remission (PR) was defined as regression by >50% of measurable lesions and absence of new lesions. Stable disease was defined as <50% regression of all measurable lesions without occurrence of new lesions. Progressive disease was defined as occurrence of new lesions or >25% growth of any initial lesion. Patients achieving unconfirmed CR without evidence of disease progression/relapse within the first 3 months of follow-up were recorded as CR. Progression-free survival was defined as the time from entering the study until documented lymphoma progression or relapse or death as a result of lymphoma. Overall survival was defined as the time from entering the study to last observation or death from any cause. Overall response (OR: CR+PR) was calculated from diagnosis or relapse for pretreated patients until death from any cause or last contact, while disease-free survival was measured from the date of CR.

Real-time PCR

Primers are shown in Supplementary Table 1. Melting curve analyses confirmed the generation of the specific PCR product expected. The PCR products were sequenced in an ABI Prism™377 DNA sequencer apparatus (PE Applied Biosystems, CA).

SUPPLEMENTARY FIGURE AND TABLES

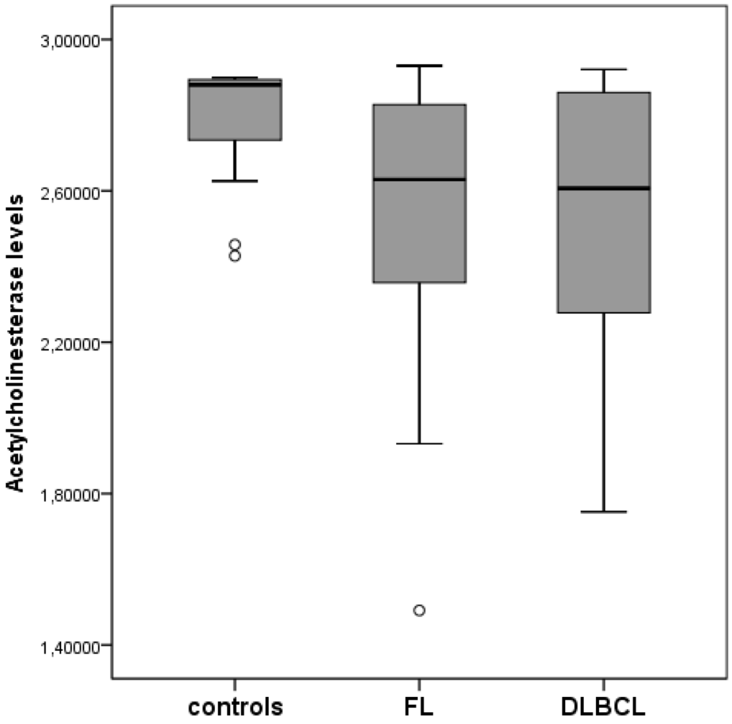

Supplementary Figure 1: Box Plots showing Acetylcholinesterase levels in the different subpopulations analyzed.

Supplementary Table 1: Clinicopathological characteristics of patients with DLBCL and FL

Characteristics	Total series(%)	DLBCL (%)	FL (%)
Stage			
I	12.4	17.0	5.6
II	27.8	39.0	11.1
III	25.8	20.3	33.3
IV	34	23.7	50.0
LNA			
<4	57.3	69.8	38.2
≥4	42.7	30.2	61.8
Bulky			
Negative	53.8	45.6	67.6
Positive	46.2	54.4	32.4
LDH levels			
Normal	71.7	67.9	79.4
High	28.3	32.1	20.6
PS			
0	32.3	19.0	55.6
1	46.9	51.7	38.9
2	16.6	22.4	5.6
3	4.2	6.9	0
IPI/FLIPI			
Low-risk (0-2)	74.2	72.9	75.0
High-risk (3-4)	25.8	27.1	25.0
1st relapse + progression			
No	82.1	81.0	82.9
Yes	17.9	19.0	17.1

LNA: number of lymph nodes affected; LDH: lactate dehydrogenase; PS: performance status

Supplementary Table 2: Comparison of PFS and OS between FL and DLBCL patients

	FL	DLBCL	*p*
	**%**	**%**	
**PFS**			
12 months	88.2% (77.4%-99%)	86.5% (77.2%-95.8%)	NS
24 months	80.7% (66.7%-94.7%)	79% (67.2%-90.8%)	
36 months	80.7% (66.7%-94.7%)	79% (67.2%-90.8%)	
**OS**			
12 months	100%	88.9% (80.5%-97.3%)	NS
24 months	92.6% (82.7%-100%)	79.9% (68.6%-91.2%)	
36 months	92.6% (82.7%-100%)	79.9% (68.6%-91.2%)	

Supplementary Table 3: Percentage of detection for each analyzed mRNA in the three subpopulations studied

		healthy controls	FL	DLBCL	*p*
C-MYC					
	absence	92.6	94.7	88.3	0.494
	presence	7.4	5.3	11.7	
BCL-6					
	absence	95.6	84.2	55.0	<0.001*/0.005**
	presence	4.4	15.8	45.0	
BCL-XL					
	absence	83.8	73.7	90.0	0.103
	presence	16.2	26.3	10.0	
NF-kB					
	absence	85.3	94.7	90.0	0.314
	presence	14.7	5.3	10.0	
PTEN					
	absence	32.4	73.7	75.0	<0.001*
	presence	67.6	26.3	25.0	
AKT					
	absence	98.5	94.7	98.3	0.425
	presence	1.5	5.3	1.7	
**N**		68	38	60	

**p* value when B-cell lymphomas were compared with healthy controls.

***p* value when DLBCL were compared with FL.

Supplementary Table 4: Number of mRNAs detected in plasma in the three subpopulations studied

number of mRNAs detected in plasma	Healthy controls	FL	DLBCL	*p*
0	13	0	0	
1	37	14	16	
2	16	16	29	<0.001
3	1	8	12	*0,408
4	1	0	3	

**p* value when DLBCL were compared with FL

Supplementary Table 5: Differences in the mRNA detection between pre- and post-treatment samples from the same patient in total series (B-cells) and subpopulations (DLBCL and FL)

	Total Series	DLBCL	FL
**C-MYC mRNA**			
No changes in both samples	74,2%	75%	73,3%
Decreased in post-treatment samples	9,7%	18,8%	0%
Increased in post-treatment samples	16,1%	6,3%	26,7%
***BCL-XL* mRNA**			
No changes in both samples	77,4%	81,3%	73,3%
Decreased in post-treatment samples	12,9%	6,3%	20%
Increased in post-treatment samples	9,7%	12,5%	6,7%
***BCL-6* mRNA**			
No changes in both samples	51,6%	37,5%	66,7%
Decreased in post-treatment samples	32,3%	43,8%	20%
Increased in post-treatment samples	16,1%	18,8%	13,3%
***NKkB* mRNA**			
No changes in both samples	93,5%	87,5%	100%
Decreased in post-treatment samples	6,5%	12,5%	0%
Increased in post-treatment samples	0%	0%	0%
***PTEN* mRNA**			
No changes in both samples	67,7%	62,5%	73,3%
Decreased in post-treatment samples	16,1%	12,5%	20%
Increased in post-treatment samples	16,1%	25%	6,7%
***AKT* mRNA**			
No changes in both samples	96,8%	100%	93,3%
Decreased in post-treatment samples	3,2%	0%	6,7%
Increased in post-treatment samples	0%	0%	0%

Supplementary Table 6: Sequences and annealing temperatures (AT) for each primer used

mRNA	Primers Sequence	AT (°C)
BCL-XL	5´AGTCGGATCGCAGCTTGG3´<br>5´TGCATTGTTCCCATAGAGTTCC3´	59
NFkB	5´ACCTCTCAGGCCCACTCGC3´<br>5´CGGTAAAGCTGAGTTTGCGG3´	59
C-MYC	5´TGGATTTTTTTCGGGTAGTGG3´F<br>5´GTCGTAGTCGAGGTCATAGTTCC3´R	59
BCL-6	5´CCTGTGAAATCTGTGGCACCCG3´F<br>5´CGCAGCTGGCTTTTGTGACGG3´R	61
PTEN	5´CAATGTTCAGTGGCGGAACTTG3´<br>5´GAACTTGTCTTCCCGTCGTGTG3´	59
AKT	5´GCAGACTGTCTCCGAGACGC3´<br>5´GCAAATGGACTGAGACGGC3´	59
